# Effect of Acacia Polyphenol Supplementation on Exercise-Induced Oxidative Stress in Mice Liver and Skeletal Muscle

**DOI:** 10.3390/antiox9010029

**Published:** 2019-12-28

**Authors:** Koichi Yada, Llion Arwyn Roberts, Natsumi Oginome, Katsuhiko Suzuki

**Affiliations:** 1 Faculty of Sport Sciences, Waseda University, Tokorozawa, Saitama 359-1192, Japan; yada_koich@yahoo.co.jp; 2 School of Allied Health Sciences, Griffith University, Gold Coast 4215, Australia; llion.roberts@griffith.edu.au; 3 School of Human Movement and Nutrition Sciences, University of Queensland, Brisbane 4072, Australia; 4 Graduate School of Sport Sciences, Waseda University, Tokorozawa, Saitama 359-1192, Japan; ognm.ntm.51@gmail.com

**Keywords:** polyphenols, antioxidants, oxidative stress, hepatotoxicity, exhaustive exercise

## Abstract

The purpose of this study was to investigate the effects of acacia polyphenol (AP) supplementation on exercise-induced oxidative stress in mouse liver and skeletal muscle. Plasma aspartate aminotransferase (AST), liver and skeletal muscle levels of thiobarbituric acid reactive substance (TBARS), and levels of skeletal muscle protein carbonyls increased immediately after exhaustive exercise. Exhaustive exercise also decreased liver glutathione (GSH). These results suggest that the exhaustive exercise used in this study induced tissue damage and oxidative stress. Contrary to our expectations, AP supplementation increased plasma AST and alanine aminotransferase activities, liver levels of TBARS, and protein carbonyls. Furthermore, AP supplementation decreased glutathione and glutathione peroxidase activity in the liver. On the other hand, AP supplementation decreased TBARS levels in skeletal muscle. These results suggest that oral high-dose AP administration decreased oxidative stress in skeletal muscle but induced oxidative stress in the liver and increased hepatotoxicity.

## 1. Introduction

Regular physical exercise or physical activity has many health benefits such as reducing the risks of cardiovascular disease, cancer, and diabetes [1,2,3]. However, acute vigorous exercise induces muscle and tissue injuries which relate to free radical and oxidative stress production [3,4]. The term oxidative stress describes a condition in which the cellular production of reactive oxygen species (ROS) exceeds the capacity of the endogenous antioxidant defense system. ROS react with cellular components including DNA, lipids, and proteins and can lead to cellular damage [5]. For regular cellular function, it is necessary to keep homeostatic conditions in the redox (oxidation–reduction) system [3,6]. Exhaustive exercise-induced tissue injury or inflammation can be called short-term oxidative stress, but insufficient antioxidant response may not be able to reverse the toxic effect of excessively produced free radicals [7]. Prolonged exercise induces antioxidant and anti-inflammatory effects on leukocyte-derived ROS, indicating a protection of oxidative damage [8]. On the other hand, another study mentioned that a single dose of antioxidant supplementation may improve endurance capacity in trained athletes [9]. Therefore, it is thought that consuming antioxidants is important for athletes and untrained individuals alike, to protect against exercise-induced oxidative stress, tissue damage, and limit decreases in exercise performance.

Acacia is an evergreen tree belonging to the genus *Acacia* in the family legume and is widely found across the Australian and African continents. The extract of Acacia catechu duramen is called gambir and has long been used as, for example, a stringent and anti-bacterial agent to treat stomatitis and diarrhea in Japan and China [10]. It has also been reported that acacia bark extract acts as an antioxidant with proven efficacy in reducing lipid peroxidation in vitro [11], while acacia has also been shown to protect against CCl_4_-induced oxidative stress in the liver and hepatic damage of rats [11]. Furthermore, Ikarashi and colleagues [10] reported that acacia polyphenol (AP) suppressed the increase of visceral white adipose tissue (WAT) mass, and the dysregulated expression of tumor necrosis factor-α and adiponectin in epididymal WAT of diabetic KKAy mice subjected to high-fat diet. Despite the aforementioned studies showing promise for AP, the effects of AP on exercise-induced oxidative stress have not been clarified. Therefore, the purpose of this study was to investigate the effects of AP supplementation on acute exercise-induced oxidative stress in mouse liver and skeletal muscle, where it is possible to elucidate the effects of AP on cell/organ compartments alongside exercise capacity which would be challenging to accomplish in healthy human models.

## 2. Methods

### 2.1. Experimental Animals and Sample Processing

Male C57BL/6J mice (9 weeks old) were purchased from Takasugi experimental animals supply (Kasukabe, Japan). Six animals were housed together in 1 cage (27 × 17 × 13 cm) in a controlled environment and light–dark cycle (lights on at 09:00 and off at 21:00). Experimental procedures followed the Guiding Principles for the Care and Use of Animals in the Waseda University Institutional Animal Care and Use Committee and were approved by the Institutional Animal Care and Use Committee of Waseda University (2015-A098). Subsequently, mice were randomly divided into 4 groups: sedentary (Sed), acacia polyphenol-supplemented (APS), exercise only (Ex), and Ex + APS groups (*n* = 6). With this design, the effects of exercise upon oxidative stress could be investigated, as well as the sole and combined effects of AP and AP and exercise on oxidative stress markers.

All mice were familiarized with treadmill running and exhaustive exercise one week before the intervention by exposure to treadmill running at 15 m/min for 10 min intervals. Mice in the APS and Ex + APS groups were fed oral AP (200 mg/kg weight) with a feeding needle one hour before the start of exhaustive exercise to allow enough time for potential AP effects to occur before exercise began (Ex + APS) or the pseudo start time (APS). We selected the dose based on previously published studies [12,13]. The AP used in this study was kindly provided by Amino Up Chemical Co., Ltd. (Sapporo, Japan). Mice in the Sed and Ex groups were fed water at the same relative time as the other groups. One hour after the AP or water supplementation, mice in the Ex and Ex + APS groups exercised on a motorized treadmill (Natsume, Kyoto, Japan) until exhaustion. The protocol used an initial speed of 18 m/min at a 5% grade for 30 min, followed by an increase of 3 m/min every 30 min until exhaustion. Exhaustion was defined as the point at which mice refused to run despite the stimulation of repeated tapping on the back of the mouse. Mice were sacrificed by isoflurane (Abbott, Tokyo, Japan) immediately after exercise (Ex and Ex + APS groups) or at the same average time after water ingestion (Sed and APS groups). Blood samples were collected from the abdominal artery, while the liver and gastrocnemius muscles were quickly excised and frozen in liquid nitrogen. Heparinized plasma was obtained by centrifuging the blood samples at 1600× *g* for 10 min at 4 °C. All tissue and plasma samples were stored at −80 °C until analysis.

The liver and skeletal muscle tissues were homogenized in tissue protein extraction reagent (T-PER; Pierce, Rockford, IL, USA) containing protease inhibitor (Complete mini protease inhibitor cocktail tablets; Roche, Mannheim, Germany) and phosphatase inhibitor (Roche, Mannheim, Germany) at 4 °C. The homogenate was subsequently centrifuged at 10,000× *g* for 15 min at 4 °C and the protein content of the supernatant was determined by a bicinchoninic acid (BCA) Protein Assay Kit (Thermo, Rockford, IL, USA) according to the manufacturer’s instructions. This supernatant was used for the measurement of markers of oxidative stress and antioxidant capacity.

### 2.2. Measurement

Plasma alanine aminotransferase (ALT) and aspartate aminotransferase (AST) activities were analyzed commercially by Oriental Yeast Co. (Tokyo, Japan). Thiobarbituric acid reactive substance (TBARS) concentrations were measured in liver and skeletal muscle tissue with a commercially available TBARS Assay Kit (Cayman Chemical Co., Ann Arbor, MI, USA) as a marker of lipid peroxidation. Protein carbonyl concentrations were measured in liver and skeletal muscle tissue with a commercially available Protein Carbonyl Assay Kit (Cayman Chemical Co., Ann Arbor, MI, USA) as a marker of protein oxidation. Glutathione (GSH) concentrations in liver and skeletal muscle tissue were determined using a commercially available GSH kit (Biooxytech GSH/GSSG-412; Oxis Health Products, Portland, OR, USA). Non-enzymatic antioxidant capacity, Trolox equivalent antioxidant capacity (TEAC), in the liver and skeletal muscle was measured according to the methods of Re et al. [14]. Finally, liver and skeletal muscle superoxide dismutase (SOD) and glutathione peroxidase (GPx) activities were measured using commercially available kits from Cayman Chemicals (Cayman Chemical Co., Ann Arbor, MI, USA). All analyses were performed with technical duplication.

### 2.3. Statistical Analysis

A two-way analysis of variance (ANOVA) was performed (SPSS V22.0, IBM Japan, Ltd., Tokyo, Japan) on the average from two technical replicates for each dependent variable to determine the main effects of exercise and/or AP supplementation. The Bonferroni post-hoc test was performed to identify differences among groups when significant main effects or interactions were present. A finding for a main effect for AP supplementation indicates that mice in both AP supplemented groups, regardless of exercise, were significantly different from non-supplemented groups. A main effect for exercise also shows a significant change due to the exhaustive exercise, regardless of AP supplementation. All data are presented as means ± standard errors (SEM), and statistical significance was accepted at *p* < 0.05.

## 3. Results

### 3.1. Running Time until Exhaustion

There was no significant difference in running time to exhaustion between the mice of the Ex group and the Ex + APS group (Ex group: 157 ± 12 min versus Ex + APS group: 146 ± 11 min, *p* = 0.521).

### 3.2. Markers of Hepatic Toxicity

**Figure 1:**
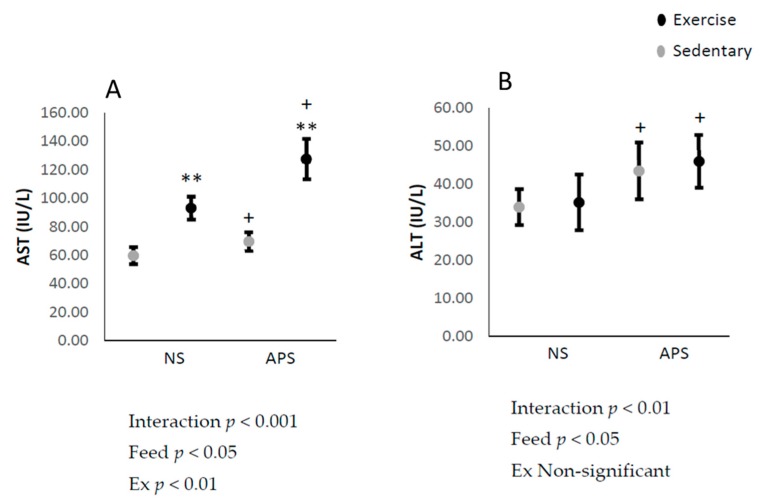
Means ± SEM plasma: (**A**) aspartate aminotransferase (AST) (control, *n* = 6; APS, *n* = 6; exercise (Ex), *n* = 4; Ex + APS, *n* = 4) and (**B**) alanine aminotransferase (ALT) (control, *n* = 6; APS, *n* = 6; Ex, *n* = 5; Ex + APS, *n* = 4). NS: non-supplemented groups, APS: acacia polyphenol-supplemented groups. ** *p* < 0.01, main effect for exercise. + *p* < 0.05, main effect for acacia polyphenol supplementation. A two-way analysis of variance (ANOVA) was performed to identify the main effect of supplement and/or exercise.

**Figure 2:**
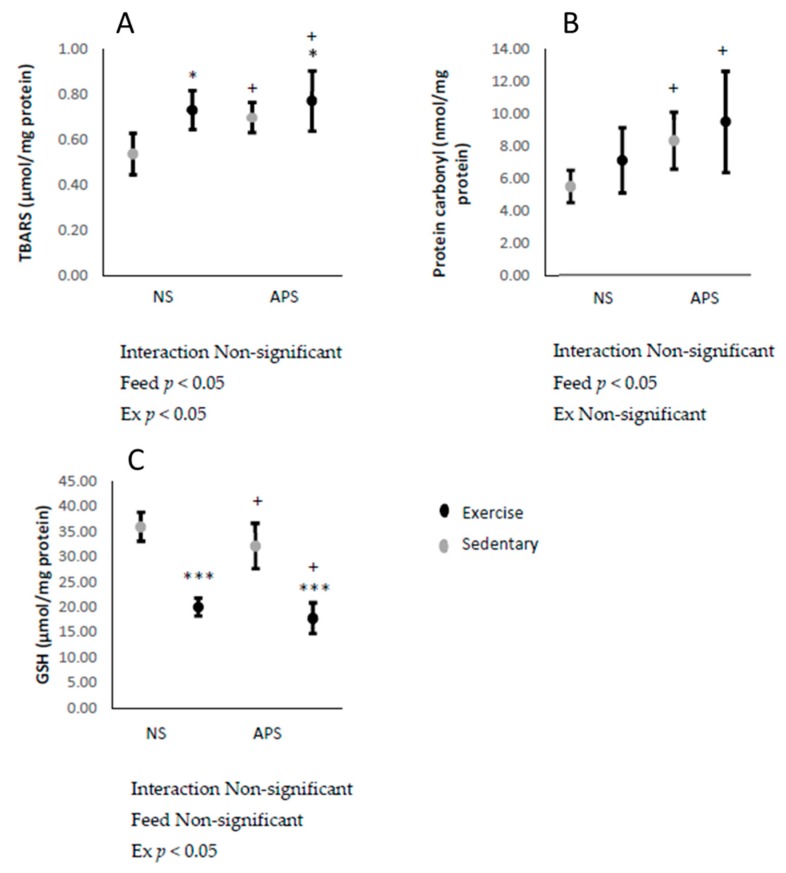
Means ± SEM: (**A**) liver thiobarbituric acid reactive substance (TBARS) (control, *n* = 6; APS, *n* = 6; Ex, *n* = 6; Ex + APS, *n* = 6), (**B**) protein carbonyl (control, *n* = 6; APS, *n* = 6; Ex, *n* = 6; Ex + APS, *n* = 6), and (**C**) glutathione (GSH) (control, *n* = 6; APS, *n* = 7; Ex, *n* = 6; Ex + APS, *n* = 6). NS: non-supplemented groups, APS: acacia polyphenol-supplemented groups. * *p* < 0.05, *** *p* < 0.001, main effect for exercise. + *p* < 0.05 main effect for acacia polyphenol supplementation. A two-way analysis of variance (ANOVA) was performed to identify the main effect of supplement and/or exercise.

Plasma AST and ALT increased after AP supplementation (*p* < 0.05; Figure 1A,B), suggesting an increase in hepatic toxicity after AP supplementation. Furthermore, AST also increased immediately after the exhaustive exercise (*p* < 0.01; Figure 2A).

### 3.3. Markers of Oxidative Stress in Liver

Liver TBARS concentrations increased immediately after the exhaustive exercise (*p* < 0.05; Figure 2A), while TBARS and protein carbonyl concentrations increased after AP supplementation (*p* < 0.05; Figure 2A,B). Furthermore, GSH decreased after exercise following AP supplementation (*p* < 0.05; Figure 2C).

### 3.4. Markers of Antioxidant Capacity in Liver

**Figure 3:**
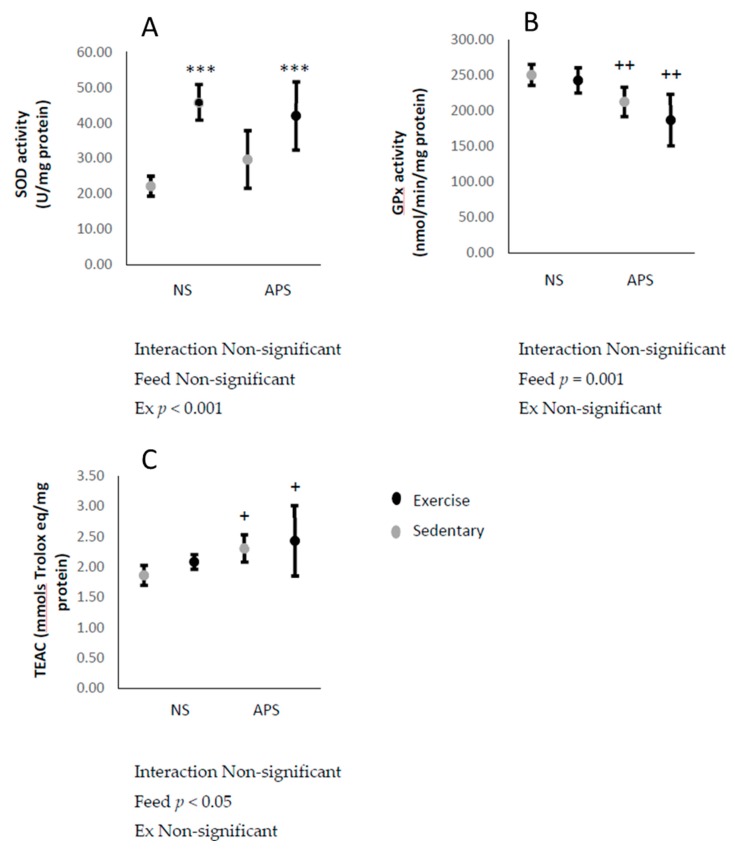
Means ± SEM: (**A**) liver superoxide dismutase (SOD) (control, *n* = 5; APS, *n* = 7; Ex, *n* = 5; Ex + APS, *n* = 6), (**B**) glutathione peroxidase (GPx) (control, *n* = 6; APS, *n* = 7; Ex, *n* = 6; Ex + APS, *n* = 6), and (**C**) Trolox equivalent antioxidant capacity (TEAC) (control, *n* = 6; APS, *n* = 7; Ex, *n* = 6; Ex + APS, *n* = 6). NS: non-supplemented groups, APS: acacia polyphenol-supplemented groups. *** *p* < 0.001, main effect for exercise. + *p* < 0.05, ++ *p* < 0.01, main effect for acacia polyphenol supplementation. A two-way analysis of variance (ANOVA) was performed to identify the main effect of supplement and/or exercise.

The antioxidant enzyme SOD activity in the liver increased immediately after the exhaustive exercise (*p* < 0.001; Figure 3A). On the other hand, liver GPx activity decreased with AP supplementation (*p* < 0.01; Figure 3B). Liver TEAC, a marker of non-enzymatic antioxidant capacity, increased with AP supplementation (*p* < 0.05; Figure 3C).

### 3.5. Markers of Oxidative Stress in Skeletal Muscle

**Figure 4:**
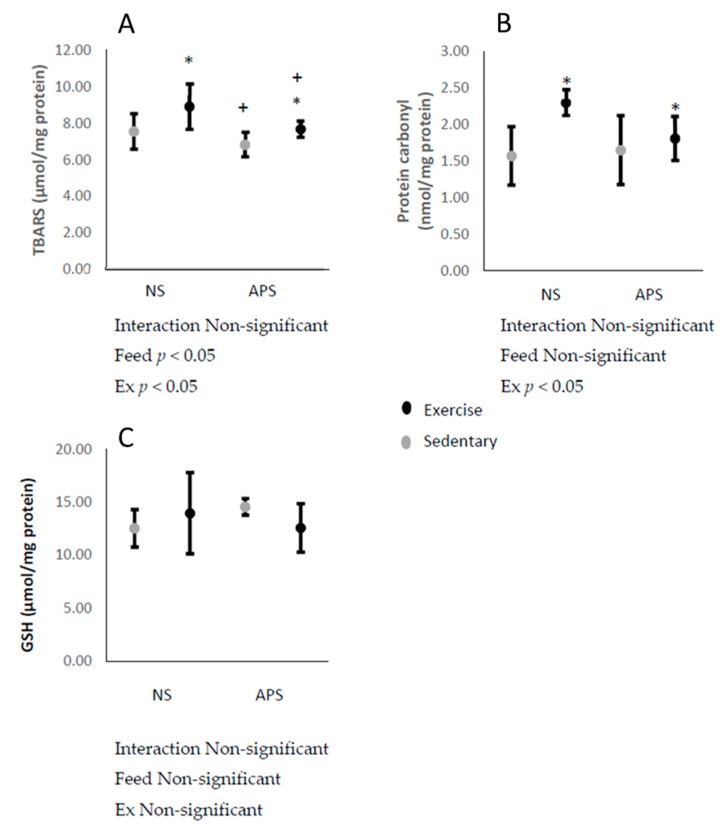
Means ± SEM: (**A**) skeletal muscle TBARS (control, *n* = 6; APS, *n* = 6; Ex, *n* = 6; Ex + APS, *n* = 5), (**B**) protein carbonyl (control, *n* = 6; APS, *n* = 5; Ex, *n* = 5; Ex + APS, *n* = 6), (**C**) and GSH (control, *n* = 6; APS, *n* = 6; Ex, *n* = 6; Ex + APS, *n* = 6). NS: non-supplemented groups, APS: acacia polyphenol-supplemented groups. * *p* < 0.05, main effect for exercise. + *p* < 0.05, main effect for acacia polyphenol supplementation. A two-way analysis of variance (ANOVA) was performed to identify the main effect of supplement and/or exercise.

Concentrations of skeletal muscle TBARS and protein carbonyls increased immediately after the exhaustive exercise (*p* < 0.05; Figure 4A,B). Skeletal muscle TBARS concentrations were decreased by AP supplementation (*p* < 0.05; Figure 4A). Skeletal muscle GSH levels were unchanged by neither exercise nor AP (Figure 4C).

### 3.6. Markers of Antioxidant Capacity in Skeletal Muscle

**Figure 5:**
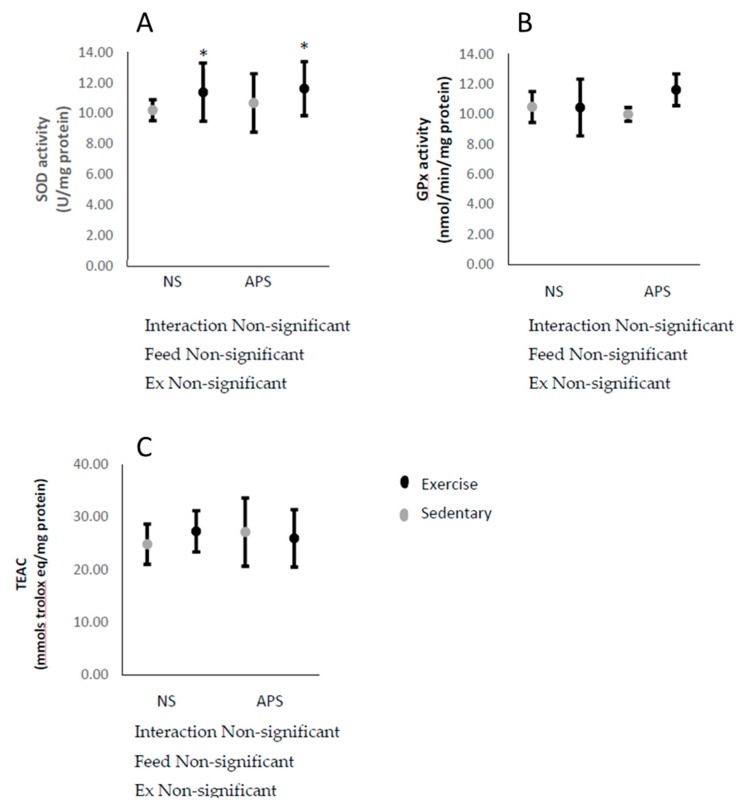
Means ± SEM: (**A**) skeletal muscle SOD (control, *n* = 6; APS, *n* = 6; Ex, *n* = 6; Ex + APS, *n* = 6), (**B**) GPx (control, *n* = 6; APS, *n* = 7; Ex, *n* = 6; Ex + APS, *n* = 6), and (**C**) TEAC (control, *n* = 6; APS, *n* = 5; Ex, *n* = 6; Ex + APS, *n* = 6). NS: non-supplemented groups, APS: acacia polyphenol-supplemented groups. * *p* < 0.05, main effect for exercise. A two-way analysis of variance (ANOVA) was performed to identify the main effect of supplement and/or exercise.

The activity of skeletal muscle SOD increased immediately after exhaustive exercise, but this response was not affected by AP (Figure 5A). Skeletal muscle GPx activity and TEAC were unchanged by neither exercise nor AP (Figure 5B,C).

## 4. Discussion

This study attempted to reveal the effects of a single dose of AP supplementation on acute exercise-induced oxidative stress in mouse liver and skeletal muscle. The main findings of this study were that AP supplementation increased oxidative stress and hepatotoxicity in the liver, whereas in contrast, AP decreased oxidative stress in skeletal muscle.

Acute strenuous exercise induces oxidative stress and/or tissue damage in several tissues including skeletal muscle, liver, and kidney [8,15,16,17,18,19]. Davies et al. [20] reported that exhaustive exercise increases ROS generation and induces tissue damage in liver and skeletal muscle. In agreement with Davies et al., we also observed that liver and skeletal muscle oxidative stress and a plasma marker of liver damage increased after exercise. It is well known that antioxidant capacity (enzymatic and non-enzymatic antioxidant activity) is increased by acute or chronic exercise [3,8,21,22,23]. For instance, SOD is known as the first line of defense of the antioxidant enzyme system; thus, we measured SOD activity in both liver and skeletal muscle. Accordingly, SOD activity increased after exercise in both liver and skeletal muscle tissues in this study. Therefore, we are content that the exercise protocol used in this study was suitable to induce skeletal muscle oxidative stress and hepatic tissue damage. However, our results did not show significant increase in muscle GPx and GSH levels, while liver GPx and GSH levels showed significant effects due to the AP administration. For such differences, we assumed that higher doses of AP may induce muscle antioxidant enzyme activity significantly.

It is known that polyphenols have powerful antioxidant capacities which is supported by much research investigating the antioxidant ability of polyphenols [24,25]. In this study, AP increased non-enzymatic antioxidant capacity in the liver. Furthermore, it has been previously reported that polyphenols induce hepatoprotective effects [26,27,28,29], while AP extracts also enhance antioxidant capacity [30,31] and induce hepatoprotective effects [32,33]. Hence, in the present study, we sought to determine the effects of AP supplementation on exercise-induced oxidative stress and tissue damage. However, contrary to our expectation, AP supplementation actually induced liver oxidative stress and hepatotoxicity, regardless of exercise. Recently, studies have reported that high doses of the phenol epigllocatehin-3-gallate (EGCG) induces hepatotoxicity and increases oxidative stress in vivo [34]. Moreover, high-dose EGCG injection has also been shown to decrease the gene expression of antioxidant enzymes of the nuclear factor erythroid 2-related factor 2 (Nrf2) pathway in the liver [35]. Therefore, it is assumed that high-dose EGCG-induced oxidative stress and hepatotoxicity is associated with the decrease of antioxidant enzyme expression. The administration of antioxidants can significantly induce Nrf2 expression [36] followed by the induction of antioxidant enzymes [37]. In accordance with these studies, we postulate that the increased hepatotoxicity, oxidative stress, and the decrease of antioxidant enzyme activity observed in the present study were induced by the dose of AP used. Further, the dose of AP supplemented in this study might have induced hepatotoxicity and oxidative stress as a result of the observed decrease in GPx activity.

On the other hand, AP supplementation decreased TBARS in the skeletal muscle but did not change the muscle’s protein carbonyls concentration. These results indicate that AP did not induce toxicity in the skeletal muscle and rather acted as an antioxidant. In support of this finding, previous studies have suggested that AP possesses an antioxidant capacity [30,31]. Increased hepatic lipid peroxidation is proportionally associated with oxidative DNA damage [38]. Furthermore, it has been reported that several polyphenols can decrease exercise-induced oxidative stress and lipid peroxidation in the skeletal muscle [39,40,41]. Consistent with these previous studies, we suggest that AP was also effective in decreasing exercise-induced oxidative stress in this study. It is thought that it is desirable to reduce the occurrence of oxidative stress in skeletal muscle, as the increase in oxidative stress leads to a decreased muscle contractile ability. In this study, we consider that our results reinforce the effectiveness of AP as an antioxidant within skeletal muscle.

Interestingly, we observed different results for oxidative stress between the liver and skeletal muscle. The ingested polyphenols enter the liver through the mesenteric vein. Subsequently, after being subjected to glucuronidation, sulfate conjugation, glutathione conjugation, etc., it is transported to the peripheral tissues to induce biological effects [42]. It is thought that oxidative stress caused by exercise was alleviated by the antioxidant power of AP that was conjugated and transferred to the peripheral tissues. On the other hand, in the liver, high AP concentrations would have rapidly resulted in increased oxidative stress and decreased GSH and GPx activity, resulting in liver injury. Here, GSH is an important intracellular component that is oxidized to glutathione disulfide (GSSG) by GPx and converted back to the reduced state by the glutathione reductase enzyme. This reaction cycle can be improved by heavy exercise [43]. Besides the liver and skeletal muscle, other organs might be also affected during acute exhaustive exercise, but our study was limited by focusing on these two major organs. It remains unclear, however, which components contained within AP acted to cause oxidative stress and liver injury which requires further investigation. Further study is also required to elucidate the effects of AP supplementation and the influence of exercise on other organs.

## 5. Conclusions

In conclusion, while supplementing with AP led to favorable antioxidant responses within the skeletal muscle, AP supplementation in fact induced hepatotoxicity and increased oxidative stress alongside decreasing GPx activity in the liver, regardless of exercise. These results suggest that oral high-dose AP administration induces oxidative stress in the liver and increases hepatotoxicity.
